# Supplementary material for: Calcium-Sensing Receptor Polymorphisms at rs1801725 Are Associated with Increased Risk of Secondary Malignancies
Source: J Pers Med. 2021 Jul 6;11(7):642. doi: 10.3390/jpm11070642 (PMC8304025; doi:10.3390/jpm11070642)
Supplement: Supplementary file 1 [file jpm-11-00642-s001.zip › CaSR_Supplementary_Materials.pdf]

Supplementary Materials

This document contains supplementary material for:

Calcium-Sensing Receptor Polymorphisms at rs1801725 Are Associated with Increased Risk of Secondary Malignancies

Ky'Era V. Actkins, Heather K. Beasley, Annika B. Faucon, Lea K. Davis and Amos M. Sakwe

Supplementary Methods and Results

Power analysis

A power analysis demonstrated that the African dataset, though smaller, remained well powered (100%) to detect the same effect sizes observed in the larger European descent sample. A standard normal distribution was assumed in the z-test family logistic regression function of G*Power 3.1.9.6. All regression analyses were performed using R 3.6.0.

Phenome-wide association study (PheWAS)

We performed a PheWAS of rs1801725 and rs1801726 as an additional secondary analysis to examine the effects of these two *CASR* SNPs across 1,372 phenotypes in the VUMC medical phenome (**Figures S1-4**). The additive and recessive models were fitted to the European and African descent datasets. Covariates included median age, sex, and the top 10 principal components for each genotyped cohort. All analyses were completed using the PheWAS 0.99.5-2 package in R 3.6.0 and additional information on the phecode mapping can be found at https://phewascatalog.org/.

Disease phenotypes associated with polymorphisms at CASR rs1801725 and rs1801726.

We performed an exploratory analysis on polymorphic variants of *CASR* at rs1801725 and rs1801726 in a PheWAS model to test if these variants were associated with calcium related disease phenotypes across the medical phenome. We show that several circulating calcium associated cancer phenotypes passed a p-value threshold of 0.05 in the additive PheWAS model (**Figure S2a**). These include secondary malignancy of respiratory organs (OR = 1.21, 95% CI = 1.08-1.35, $p = 9.32 \times 10^{-4}$), secondary malignancy of bone (OR = 1.20, 95% CI = 1.06-1.36, $p = 4.24 \times 10^{-3}$), and hypercalcemia (OR = 1.22, 95% CI = 1.06-1.40, $p = 5.53 \times 10^{-3}$). Using a recessive PheWAS model, we show that polymorphisms at rs1801725 were significantly (p < 3.64

x 10^{-5}) associated with deficiency of humoral immunity (OR = 1.21, 95% CI = 1.08-1.35, p = 9.32 x 10^{-4}) in the European dataset (**Figures S1a**). We also demonstrate that polymorphisms at this locus are nominally associated ($p < 0.05$) with bone-related cancers (secondary malignancy of bone and cancer of bone and connective tissues) and lymphomas (non-Hodgkin's, acute lymphoid and chronic lymphoid leukemias).

Phenome-wide significant effects were not observed in the African dataset for either of the CaSR SNPs nor were there any nominal associations with cancer phenotypes. However, we observed a modest association between rs1801275 and osteoporosis, osteopenia, and pathological fracture (OR = 1.41, 95% CI = 1.05-1.90, p = 0.02) (**Figures S3a and S4a**).

Table S1. Description of cancer phecodes used in this study.

Primary Cancer Phecodes	
**Phecode**	**Phenotype**
145	Cancer of mouth
149	Cancer of larynx, pharynx, nasal cavities
150	Cancer of esophagus
151	Cancer of stomach
153	Colorectal cancer
155	Cancer of liver and intrahepatic bile duct
157	Pancreatic cancer
164	Cancer of intrathoracic organs
165	Cancer within the respiratory system
170	Cancer of bone and connective tissue
172	Skin cancer
174	Breast cancer
180	Cervical cancer and dysplasia
184	Cancer of other female genital organs
185	Cancer of prostate
187	Cancer of other male genital organs
189	Cancer of urinary organs (incl. kidney and bladder)
190	Cancer of eye
193	Thyroid cancer
194	Cancer of other endocrine glands
195	Cancer suspected or other
202	Cancer of other lymphoid histiocytic tissue
**Secondary Malignancy Phecodes**	
**Phecode**	**Phenotype**
198.1	Secondary malignancy of lymph nodes
198.2	Secondary malignancy of respiratory organs
198.3	Secondary malignant neoplasm of digestive systems
198.4	Secondary malignant neoplasm of liver
198.5	Secondary malignancy of brain/spine
198.6	Secondary malignancy of bone
198.7	Secondary malignant neoplasm of skin

Note: Phecode information was obtained from https://phewascatalog.org/.

Table S2. Relationship between circulating total calcium and primary cancer diagnosis at various pathological sites.

Phenotype	Estimate	SE	Z Value	P	LCI	OR	UCI	Controls	Cases	N
Cancer of mouth	-0.27	0.02	-11.09	1.33E-28	0.73	0.77	0.80	459696	2089	461785
Cancer of larynx	-0.27	0.02	-13.88	8.45E-44	0.73	0.76	0.79	459696	3170	462866
Cancer of esophagus	-0.62	0.04	-14.22	6.57E-46	0.49	0.54	0.59	441785	608	442393
Cancer of stomach	-0.60	0.04	-15.12	1.13E-51	0.51	0.55	0.59	441785	751	442536
Colorectal cancer	-0.20	0.02	-12.58	2.88E-36	0.79	0.82	0.84	420360	4677	425037
Cancer of liver and intrahepatic bile duct	-0.57	0.03	-20.30	1.30E-91	0.54	0.57	0.60	441785	1501	443286
Pancreatic cancer	-0.61	0.03	-18.30	8.55E-75	0.51	0.55	0.58	441785	1078	442863
Cancer of intrathoracic organs	-0.21	0.07	-3.17	1.51E-03	0.71	0.81	0.92	459911	281	460192
Cancer within the respiratory system	-0.18	0.02	-11.12	9.83E-29	0.81	0.83	0.86	459911	4594	464505
Cancer of bone and connective tissue	-0.36	0.02	-18.33	5.18E-75	0.67	0.70	0.72	462114	3098	465212
Skin cancer	0.21	0.01	24.83	4.79E-136	1.21	1.23	1.25	418630	18368	436998
Breast cancer	0.23	0.01	20.25	3.84E-91	1.23	1.26	1.29	432011	9672	441683
Cervical cancer and dysplasia	-0.17	0.02	-10.27	9.28E-25	0.82	0.84	0.87	423209	4496	427705
Cancer of other female genital organs	-0.13	0.03	-4.94	7.99E-07	0.84	0.88	0.93	439272	1914	441186
Cancer of prostate	0.17	0.01	13.33	1.58E-40	1.15	1.18	1.21	431562	8155	439717
Cancer of other male genital organs	0.07	0.04	1.72	0.09	0.99	1.07	1.16	455311	747	456058
Cancer of urinary organs (incl. kidney and bladder)	-0.03	0.01	-2.36	0.02	0.94	0.97	0.99	459109	5906	465015
Cancer of eye	0.04	0.05	0.71	0.48	0.94	1.04	1.15	457010	425	457435
Thyroid cancer	-0.17	0.02	-8.52	1.60E-17	0.81	0.84	0.87	456165	2971	459136
Cancer of other endocrine glands	-0.26	0.05	-5.00	5.88E-07	0.70	0.77	0.85	456165	452	456617
Cancer suspected or other	-0.28	0.01	-23.18	7.06E-119	0.74	0.75	0.77	400880	8270	409150
Cancer of other lymphoid	-0.18	0.02	-11.26	2.12E-29	0.81	0.84	0.86	450076	4921	454997

Logistic regression models were performed on cancer phenotypes and age adjusted inverse normalized calcium levels. The models were adjusted for sex and race. Significant results that passed Bonferroni correction ($p < 3.38 \times 10^{-4}$) are highlighted. LCI = lower confidence interval; OR = odds ratio; UCI = upper confidence interval.

Table S3. Relationship between circulating ionized calcium and primary cancer diagnosis at various pathological sites.

Phenotype	Estimate	SE	Z Value	P	LCI	OR	UCI	Controls	Cases	N
Cancer of mouth	-0.12	0.03	-3.54	3.98E-04	0.83	0.89	0.95	81125	1020	82145
Cancer of larynx	-0.13	0.03	-4.64	3.57E-06	0.83	0.88	0.93	81125	1415	82540
Cancer of esophagus	-0.10	0.06	-1.80	0.07	0.81	0.90	1.01	74622	358	74980
Cancer of stomach	-0.11	0.05	-2.21	0.03	0.81	0.89	0.99	74622	414	75036
Colorectal cancer	-0.18	0.03	-7.09	1.38E-12	0.80	0.84	0.88	72383	1731	74114
Cancer of liver and intrahepatic bile duct	0.18	0.04	4.80	1.55E-06	1.11	1.19	1.28	74622	828	75450
Pancreatic cancer	-0.23	0.04	-5.36	8.33E-08	0.73	0.79	0.86	74622	576	75198
Cancer of intrathoracic organs	0.02	0.09	0.19	0.85	0.86	1.02	1.20	80752	148	80900
Cancer within the respiratory system	0.01	0.02	0.25	0.80	0.96	1.01	1.05	80752	1955	82707
Cancer of bone and connective tissue	-0.21	0.03	-7.70	1.35E-14	0.77	0.81	0.86	81401	1515	82916
Skin cancer	0.07	0.02	4.33	1.47E-05	1.04	1.07	1.11	74506	4215	78721
Breast cancer	0.02	0.02	0.75	0.45	0.97	1.02	1.07	77908	1765	79673
Cervical cancer and dysplasia	-0.03	0.04	-0.61	0.54	0.90	0.98	1.06	77164	612	77776
Cancer of other female genital organs	-0.29	0.04	-7.16	8.16E-13	0.69	0.74	0.81	78633	612	79245
Cancer of prostate	-0.19	0.03	-7.70	1.31E-14	0.78	0.82	0.87	74796	1919	76715
Cancer of other male genital organs	-0.13	0.08	-1.63	0.10	0.76	0.88	1.03	80719	195	80914
Cancer of urinary organs (incl. kidney and bladder)	-0.29	0.02	-14.11	3.17E-45	0.72	0.75	0.78	80201	2833	83034
Cancer of eye	-0.12	0.09	-1.27	0.20	0.74	0.89	1.07	79423	121	79544
Thyroid cancer	-0.20	0.03	-7.09	1.30E-12	0.78	0.82	0.87	78983	1434	80417
Cancer of other endocrine glands	-0.33	0.06	-5.29	1.23E-07	0.64	0.72	0.81	78983	280	79263
Cancer suspected or other	-0.11	0.02	-6.44	1.22E-10	0.87	0.90	0.93	62274	3926	66200
Cancer of other lymphoid	0.03	0.03	1.25	0.21	0.98	1.03	1.09	77518	1655	79173

Logistic regression models were performed on primary cancer phenotypes and age adjusted inverse normalized ionized calcium levels. The models were adjusted for sex and race. Significant results that passed Bonferroni correction ($p < 3.38 \times 10^{-4}$) are highlighted. LCI = lower confidence interval; OR = odds ratio; UCI = upper confidence interval.

Table S4. Relationship between circulating vitamin D and primary cancer diagnosis at various pathological sites.

Phenotype	Estimate	SE	Z Value	P	LCI	OR	UCI	Controls	Cases	N
Cancer of mouth	-0.02	0.06	-0.35	0.73	0.87	0.98	1.10	103713	336	104049
Cancer of larynx	-0.01	0.05	-0.13	0.90	0.90	0.99	1.09	103713	485	104198
Cancer of esophagus	-0.36	0.10	-3.66	2.49E-04	0.58	0.70	0.85	97392	124	97516
Cancer of stomach	-0.38	0.08	-4.94	7.95E-07	0.59	0.68	0.80	97392	196	97588
Colorectal cancer	-0.13	0.03	-4.65	3.26E-06	0.83	0.88	0.93	85915	1517	87432
Cancer of liver and intrahepatic bile duct	-0.14	0.05	-3.05	2.26E-03	0.79	0.87	0.95	97392	551	97943
Pancreatic cancer	-0.37	0.07	-5.43	5.78E-08	0.61	0.69	0.79	97392	253	97645
Cancer of intrathoracic organs	-0.09	0.13	-0.73	0.47	0.71	0.91	1.17	103987	67	104054
Cancer within the respiratory system	-0.26	0.04	-7.15	8.55E-13	0.72	0.77	0.83	103987	867	104854
Cancer of bone and connective tissue	-0.08	0.05	-1.67	0.09	0.84	0.92	1.01	104414	513	104927
Skin cancer	0.06	0.01	4.25	2.17E-05	1.03	1.06	1.09	88268	6219	94487
Breast cancer	0.12	0.02	7.44	9.89E-14	1.10	1.13	1.17	92103	4237	96340
Cervical cancer and dysplasia	-0.05	0.03	-1.64	0.10	0.90	0.95	1.01	89766	1383	91149
Cancer of other female genital organs	-0.05	0.05	-1.03	0.30	0.87	0.95	1.04	95568	531	96099
Cancer of prostate	0.05	0.03	1.53	0.13	0.99	1.05	1.11	96306	1453	97759
Cancer of other male genital organs	-0.12	0.10	-1.27	0.20	0.74	0.89	1.07	103108	135	103243
Cancer of urinary organs (incl. kidney and bladder)	-0.24	0.03	-7.77	7.75E-15	0.74	0.79	0.84	103536	1223	104759
Cancer of eye	-0.41	0.12	-3.26	1.10E-03	0.52	0.67	0.85	102748	72	102820
Thyroid cancer	0.16	0.03	5.21	1.92E-07	1.10	1.17	1.25	101088	1199	102287
Cancer of other endocrine glands	-0.06	0.09	-0.64	0.52	0.78	0.94	1.13	101088	128	101216
Cancer suspected or other	-0.20	0.02	-8.41	4.18E-17	0.79	0.82	0.86	84362	2185	86547
Cancer of other lymphoid	-0.11	0.03	-4.03	5.56E-05	0.84	0.89	0.94	99708	1452	101160

Logistic regression models were performed on primary cancers and age adjusted inverse normalized vitamin D levels. The models were adjusted for sex and race. Significant results that passed Bonferroni correction ($p < 3.38 \times 10^{-4}$) are highlighted. LCI = lower confidence interval; OR = odds ratio; UCI = upper confidence interval.

Table S5. Relationship between circulating intact parathyroid hormone and primary cancer diagnosis at various pathological sites.

Phenotype	Estimate	SE	Z Value	P	LCI	OR	UCI	Controls	Cases	N
Cancer of mouth	-0.25	0.08	-3.08	2.08E-03	0.66	0.78	0.91	33928	156	34084
Cancer of larynx	-0.33	0.07	-4.79	1.64E-06	0.63	0.72	0.82	33928	217	34145
Cancer of esophagus	-0.14	0.14	-0.99	0.32	0.66	0.87	1.14	31427	53	31480
Cancer of stomach	0.20	0.11	1.79	0.07	0.98	1.23	1.53	31427	83	31510
Colorectal cancer	-0.04	0.05	-0.76	0.45	0.87	0.96	1.06	28218	451	28669
Cancer of liver and intrahepatic bile duct	-0.08	0.09	-0.87	0.38	0.78	0.93	1.10	31427	132	31559
Pancreatic cancer	0.10	0.09	1.07	0.28	0.92	1.10	1.32	31427	131	31558
Cancer of intrathoracic organs	-0.07	0.18	-0.37	0.71	0.66	0.94	1.34	33946	33	33979
Cancer within the respiratory system	-0.18	0.05	-3.55	3.93E-04	0.76	0.84	0.92	33946	437	34383
Cancer of bone and connective tissue	-0.26	0.07	-3.93	8.48E-05	0.68	0.77	0.88	34225	251	34476
Skin cancer	-0.06	0.02	-2.63	8.66E-03	0.91	0.94	0.99	28611	2511	31122
Breast cancer	-0.16	0.03	-4.91	9.26E-07	0.80	0.85	0.91	30365	1216	31581
Cervical cancer and dysplasia	0.12	0.05	2.20	0.03	1.01	1.13	1.25	29889	409	30298
Cancer of other female genital organs	-0.01	0.07	-0.14	0.89	0.86	0.99	1.14	31420	219	31639
Cancer of prostate	-0.06	0.04	-1.51	0.13	0.87	0.94	1.02	30910	648	31558
Cancer of other male genital organs	-0.01	0.13	-0.07	0.94	0.77	0.99	1.27	33601	62	33663
Cancer of urinary organs (incl. kidney and bladder)	0.22	0.03	6.79	1.16E-11	1.17	1.25	1.33	33151	1034	34185
Cancer of eye	0.15	0.17	0.87	0.38	0.83	1.16	1.62	33678	39	33717
Thyroid cancer	-0.76	0.03	-22.82	2.76E-115	0.44	0.47	0.50	31033	1128	32161
Cancer of other endocrine glands	0.18	0.12	1.42	0.16	0.93	1.19	1.52	31033	70	31103
Cancer suspected or other	-0.22	0.03	-6.31	2.73E-10	0.75	0.80	0.86	25980	949	26929
Cancer of other lymphoid	-0.13	0.04	-3.00	2.71E-03	0.81	0.88	0.96	32164	590	32754

Logistic regression models were performed on primary cancers and age adjusted inverse normalized parathyrin intact levels. The models were adjusted for sex and race. Significant results that passed Bonferroni correction ($p < 3.38 \times 10^{-4}$) are highlighted. LCI = lower confidence interval; OR = odds ratio; UCI = upper confidence interval.

Table S6. Association of circulating calcium, vitamin D and parathyroid hormone with secondary malignancies.

Phenotype	Estimate	SE	Z Value	P	LCI	OR	UCI	Phecode	Controls	Cases	N
**Calcium**											
Secondary malignancy of lymph nodes	-0.13	0.01	-9.34	9.48E-21	0.86	0.88	0.90	400880	6716	407596	-0.13
Secondary malignancy of respiratory organs	-0.23	0.02	-12.81	1.45E-37	0.77	0.79	0.82	400880	3759	404639	-0.23
Secondary malignant neoplasm of digestive systems	-0.50	0.03	-19.24	1.63E-82	0.58	0.61	0.64	400880	1779	402659	-0.50
Secondary malignant neoplasm of liver	-0.37	0.02	-18.24	2.72E-74	0.66	0.69	0.72	400880	2926	403806	-0.37
Secondary malignancy of brain/spine	-0.29	0.03	-11.21	3.70E-29	0.71	0.75	0.79	400880	1794	402674	-0.29
Secondary malignancy of bone	-0.26	0.02	-13.90	6.09E-44	0.75	0.77	0.80	400880	3530	404410	-0.26
Secondary malignant neoplasm of skin	-0.07	0.04	-1.71	0.09	0.86	0.93	1.01	400880	725	401605	-0.07
**Ionized Calcium**											
Secondary malignancy of lymph nodes	-0.17	0.02	-8.22	2.00E-16	0.81	0.85	0.88	62274	2740	65014	-0.17
Secondary malignancy of respiratory organs	-0.14	0.02	-5.75	9.17E-09	0.83	0.87	0.91	62274	1764	64038	-0.14
Secondary malignant neoplasm of digestive systems	-0.32	0.03	-9.65	5.06E-22	0.68	0.73	0.78	62274	988	63262	-0.32
Secondary malignant neoplasm of liver	-0.19	0.03	-6.57	5.01E-11	0.78	0.83	0.88	62274	1328	63602	-0.19
Secondary malignancy of brain/spine	-0.20	0.03	-5.82	5.98E-09	0.77	0.82	0.88	62274	947	63221	-0.20
Secondary malignancy of bone	-0.08	0.03	-3.25	1.17E-03	0.88	0.92	0.97	62274	1656	63930	-0.08
Secondary malignant neoplasm of skin	-0.11	0.06	-1.74	0.08	0.79	0.90	1.01	62274	267	62541	-0.11
**Vitamin D**											
Secondary malignancy of lymph nodes	-0.05	0.03	-1.83	0.07	0.91	0.96	1.00	84362	1851	86213	-0.05
Secondary malignancy of respiratory organs	-0.29	0.04	-6.89	5.45E-12	0.69	0.75	0.82	84362	675	85037	-0.29
Secondary malignant neoplasm of digestive systems	-0.33	0.06	-5.82	5.83E-09	0.64	0.72	0.80	84362	361	84723	-0.33
Secondary malignant neoplasm of liver	-0.33	0.04	-7.57	3.84E-14	0.66	0.72	0.78	84362	605	84967	-0.33
Secondary malignancy of brain/spine	-0.27	0.06	-4.24	2.28E-05	0.67	0.76	0.86	84362	283	84645	-0.27
Secondary malignancy of bone	-0.18	0.04	-4.58	4.66E-06	0.77	0.84	0.90	84362	748	85110	-0.18
Secondary malignant neoplasm of skin	-0.30	0.10	-3.16	1.58E-03	0.61	0.74	0.89	84362	125	84487	-0.30

Parathyroid Hormone											
Secondary malignancy of lymph nodes	-0.33	0.04	-8.61	7.09E-18	0.67	0.72	0.78	25980	790	26770	-0.33
Secondary malignancy of respiratory organs	-0.25	0.05	-4.61	4.00E-06	0.70	0.78	0.87	25980	379	26359	-0.25
Secondary malignant neoplasm of digestive systems	-0.16	0.08	-2.04	0.04	0.73	0.85	0.99	25980	180	26160	-0.16
Secondary malignant neoplasm of liver	-0.24	0.06	-3.90	9.68E-05	0.70	0.79	0.89	25980	305	26285	-0.24
Secondary malignancy of brain/spine	-0.38	0.09	-4.27	1.96E-05	0.57	0.68	0.81	25980	141	26121	-0.38
Secondary malignancy of bone	-0.38	0.05	-7.22	5.26E-13	0.62	0.68	0.76	25980	396	26376	-0.38
Secondary malignant neoplasm of skin	-0.31	0.12	-2.54	0.01	0.58	0.74	0.93	25980	79	26059	-0.31

The logistic regressions models were adjusted for sex, median age, and principal components 1-10. Significant results that passed Bonferroni correction ($p < 3.38 \times 10^{-4}$) are highlighted. LCI = lower confidence interval; OR = odds ratio; UCI = upper confidence interval.

Table S7. Associating of calcium, vitamin D and parathyroid hormone levels with polymorphisms at rs1801725 and rs1801726 in individuals of European descent.

	rs1801725				rs1801726			
	All	0 RA	1 RA	2 RA	All	0 RA	1 RA	2 RA
Calcium (mg/dL)								
N	51171	38094	12131	946	51171	47415	3675	79
Median [IQR]	9.30 [9.05, 9.60]	9.30 [9.00, 9.60]	9.40 [9.10, 9.60]	9.50 [9.20, 9.70]	9.30 [9.05, 9.60]	9.30 [9.05, 9.60]	9.30 [9.00, 9.60]	9.40 [9.03, 9.70]
Ionized Calcium (mg/dL)								
N	13897	10369	3281	247	13897	12866	1008	22
Median [IQR]	4.52 [4.32, 4.73]	4.51 [4.31, 4.73]	4.55 [4.35, 4.77]	4.59 [4.34, 4.79]	4.52 [4.32, 4.73]	4.52 [4.32, 4.73]	4.53 [4.33, 4.74]	4.52 [4.43, 4.69]
Vitamin D (ng/mL)								
N	18883	14076	4452	355	18883	17497	1354	31
Median [IQR]	31.00 [24.00, 39.00]	31.00 [24.00, 39.00]	31.00 [24.00, 39.00]	32.00 [24.00, 39.75]	31.00 [24.00, 39.00]	31.00 [24.00, 39.00]	31.00 [24.00, 39.00]	28.00 [21.50, 43.25]
Parathyroid Hormone (pg/mL)								
N	6936	5133	1659	144	6936	6430	497	9
Median [IQR]	56.00 [36.00, 91.00]	56.00 [36.00, 91.00]	56.00 [35.00, 88.25]	59.50 [39.75, 106.25]	56.00 [36.00, 91.00]	56.00 [36.00, 91.00]	55.50 [36.00, 88.50]	46.00 [39.00, 205.00]

Median (a) calcium, (b) ionized calcium, (c) vitamin D, and (d) parathyroid hormone levels. Wilcoxon rank sum test was used to determine statistical significance between genotypes ($p < 0.05$). RA = risk alleles, IQR = interquartile range.

Table S8. Associating of calcium, vitamin D and parathyroid hormone levels with polymorphisms at rs1801725 and rs1801726 in individuals of African descent.

	rs1801725				rs1801726			
	All	0 RA	1 RA	2 RA	All	0 RA	1 RA	2 RA
Calcium (mg/dL)								
N	9878	9159	694	18	9878	6837	2749	283
Median [IQR]	9.40 [9.10, 9.60]	9.40 [9.10, 9.60]	9.40 [9.20, 9.70]	9.43 [9.22, 9.88]	9.40 [9.10, 9.60]	9.40 [9.10, 9.60]	9.40 [9.10, 9.60]	9.40 [9.10, 9.60]
Ionized Calcium (mg/dL)								
N	2336	2175	156	4	2336	1561	709	66
Median [IQR]	4.57 [4.35, 4.80]	4.57 [4.34, 4.80]	4.65 [4.45, 4.89]	4.53 [4.41, 4.79]	4.57 [4.35, 4.80]	4.57 [4.35, 4.80]	4.57 [4.33, 4.81]	4.55 [4.33, 4.77]
Vitamin D (ng/mL)								
N	3341	3115	219	4	3341	2289	924	124
Median [IQR]	23.00 [17.00, 30.50]	23.00 [16.50, 30.50]	24.00 [18.00, 31.00]	19.50 [18.25, 22.25]	23.00 [17.00, 30.50]	22.50 [16.50, 30.00]	23.00 [16.88, 31.00]	24.75 [18.90, 31.25]
Parathyroid Hormone (pg/mL)								
N	1430	1343	86	1	1430	976	408	45
Median [IQR]	82.00 [50.0, 160.0]	82.00 [49.0, 160.3]	87.25 [59.6, 151.4]	49.00 [49.00, 49.00]	82.00 [50.00, 160.00]	82.75 [50.4, 159.25]	80.50 [48.00, 158.1]	89.00 [52.00, 167.00]

Median (a) calcium, (b) ionized calcium, (c) vitamin D, and (d) parathyroid hormone levels. Wilcoxon rank sum test was used to determine statistical significance between genotypes ($p < 0.05$). RA = risk alleles, IQR = interquartile range.

Table S9. Relationship between CaSR SNPs and circulating calcium, vitamin D and parathyroid hormone levels.

Lab	Estimate	SE	T Value	P	LCI	OR	UCI	0 RA	1 RA	2 RA
European Genotyped Sample: rs1801725										
Calcium	0.14	0.01	18.08	8.40E-73	1.13	1.15	1.16	38098	12132	946
Ionized Calcium	0.10	0.02	6.06	1.43E-09	1.07	1.10	1.14	10370	3282	247
Vitamin D	-0.01	0.01	-0.67	0.50	0.96	0.99	1.02	14078	4452	355
Parathyroid Hormone	0.001	0.02	0.03	0.98	0.95	1.00	1.05	5134	1659	144
European Genotyped Sample: rs1801726										
Calcium	-0.02	0.01	-1.41	0.16	0.96	0.98	1.01	47420	3675	79
Ionized Calcium	0.03	0.03	1.07	0.28	0.97	1.03	1.09	12868	1008	22
Vitamin D	-0.02	0.03	-0.66	0.51	0.94	0.98	1.03	17499	1354	31
Parathyroid Hormone	0.03	0.04	0.57	0.57	0.94	1.03	1.12	6431	497	9
African Descent Genotyped Sample: rs1801725										
Calcium	0.12	0.03	3.68	2.34E-04	1.06	1.12	1.20	9159	694	18
Ionized Calcium	0.16	0.08	1.98	4.73E-02	1.00	1.17	1.37	2175	156	4
Vitamin D	0.07	0.07	1.08	0.28	0.94	1.07	1.22	3115	219	4
Parathyroid Hormone	0.10	0.11	0.94	0.35	0.90	1.11	1.37	1343	86	1
African Descent Genotyped Sample: rs1801726										
Calcium	0.002	0.02	0.13	0.90	0.97	1.00	1.03	6837	2749	283
Ionized Calcium	0.01	0.04	0.36	0.72	0.94	1.01	1.09	1561	709	66
Vitamin D	0.06	0.03	1.83	0.07	1.00	1.06	1.12	2289	924	124
Parathyroid Hormone	0.03	0.05	0.64	0.52	0.94	1.03	1.13	976	408	45

Laboratory values were inverse normalized and adjusted for age. The regression models were adjusted for principal components 1-10. Significant results that passed Bonferroni correction ($p < 3.38 \times 10^{-4}$) are highlighted. LCI = lower confidence interval; OR = odds ratio; UCI = upper confidence interval; RA = risk alleles.

Table S10. Relationship between circulating calcium, vitamin D and parathyroid hormone levels in CaSR rs1801725 allele carriers before and after cancer diagnosis.

Lab	Estimate	SE	T Value	P	LCI	OR	UCI	0 RA	1 RA	2 RA
European Genotyped Sample – Before Cancer Diagnosis Labs rs1801725										
Calcium	0.13	0.01	17.79	1.53E-70	1.13	1.14	1.16	36807	11746	917
Ionized Calcium	0.09	0.02	5.24	1.66E-07	1.06	1.09	1.13	8948	2813	219
Vitamin D	-0.01	0.01	-0.86	0.39	0.96	0.99	1.02	12937	4106	330
Parathyroid Hormone	-0.02	0.02	-0.64	0.52	0.94	0.98	1.03	4605	1495	126
European Genotyped Sample – After Cancer Diagnosis Labs rs1801725										
Calcium	0.12	0.02	5.31	1.16E-07	1.08	1.12	1.17	5351	1688	126
Ionized Calcium	0.09	0.02	5.24	1.66E-07	1.06	1.09	1.13	8948	2813	219
Vitamin D	0.02	0.04	0.58	0.56	0.94	1.02	1.11	1707	547	37
Parathyroid Hormone	0.03	0.06	0.50	0.62	0.92	1.03	1.16	694	234	20

Logistic regressions models were performed on CaSR rs1801725 and stratified laboratory values based on the first diagnosis of a primary cancer for European descent individuals. Laboratory values were inverse normalized and adjusted for age and the regression models were adjusted for principal components 1-10. Significant results that passed Bonferroni correction ($p < 3.38 \times 10^{-4}$) highlighted. LCI = lower confidence interval; OR = odds ratio; UCI = upper confidence interval; RA = risk alleles.

Table S11. Association of CaSR SNPs with secondary malignancies in European descent individuals – Additive regression analysis.

Phenotype	Estimate	SE	Z Value	P	LCI	OR	UCI	Controls	Cases	0 Alleles	1 Allele	2 Alleles
rs1801725												
Secondary malignancy of lymph nodes	0.08	0.05	1.81	0.07	0.99	1.09	1.19	40476	2179	31719	10150	786
Secondary malignancy of respiratory organs	0.19	0.06	3.30	9.61E-04	1.08	1.21	1.35	40476	1249	31003	9958	764
Secondary malignant neoplasm of digestive systems	0.07	0.08	0.79	0.43	0.91	1.07	1.26	40476	615	30570	9774	747
Secondary malignant neoplasm of liver	0.11	0.07	1.73	0.08	0.99	1.12	1.28	40476	955	30808	9869	754
Secondary malignancy of brain/spine	-0.01	0.09	-0.13	0.89	0.83	0.99	1.18	40476	547	30530	9749	744
Secondary malignancy of bone	0.18	0.06	2.86	4.28E-03	1.06	1.20	1.36	40476	958	30806	9863	765
Secondary malignant neoplasm of skin	0.23	0.12	1.91	0.06	0.99	1.26	1.60	40476	259	30301	9693	741
rs1801726												
Secondary malignancy of lymph nodes	0.02	0.08	0.27	0.79	0.87	1.02	1.20	40476	2179	39517	3068	70
Secondary malignancy of respiratory organs	0.06	0.10	0.60	0.55	0.87	1.07	1.31	40476	1249	38655	3001	69
Secondary malignant neoplasm of digestive systems	-0.01	0.15	-0.07	0.94	0.73	0.99	1.33	40476	615	38072	2953	66
Secondary malignant neoplasm of liver	-0.13	0.13	-1.01	0.31	0.68	0.88	1.13	40476	955	38396	2967	68
Secondary malignancy of brain/spine	-0.08	0.17	-0.50	0.62	0.66	0.92	1.27	40476	547	38014	2941	68
Secondary malignancy of bone	-0.09	0.13	-0.69	0.49	0.71	0.92	1.17	40476	958	38397	2968	69
Secondary malignant neoplasm of skin	0.16	0.21	0.73	0.47	0.77	1.17	1.78	40476	259	37740	2928	67

The models were adjusted for median age, sex, and principal components 1-10. Results that passed a significance threshold of $p < 0.01$ are highlighted. None of the results passed Bonferroni correction ($p < 3.38 \times 10^{-4}$). LCI = lower confidence interval; OR = odds ratio; UCI = upper confidence interval.

Table S12. Association of CaSR SNPs with secondary malignancies in European descent individuals – Recessive regression analysis

Phenotype	Estimate	SE	Z Value	P	LCI	OR	UCI	Controls	Cases	0 Alleles	1 Allele
				rs1801725							
Secondary malignancy of lymph nodes	0.30	0.15	2.04	0.04	1.01	1.36	1.82	40476	2179	41869	786
Secondary malignancy of respiratory organs	0.29	0.19	1.49	0.14	0.91	1.33	1.95	40476	1249	40961	764
Secondary malignant neoplasm of digestive systems	0.10	0.30	0.33	0.74	0.62	1.10	1.97	40476	615	40344	747
Secondary malignant neoplasm of liver	0.12	0.24	0.52	0.61	0.71	1.13	1.80	40476	955	40677	754
Secondary malignancy of brain/spine	-0.05	0.34	-0.15	0.88	0.49	0.95	1.85	40476	547	40279	744
Secondary malignancy of bone	0.60	0.19	3.12	1.80E-03	1.25	1.82	2.65	40476	958	40669	765
Secondary malignant neoplasm of skin	0.32	0.42	0.77	0.44	0.61	1.38	3.13	40476	259	39994	741
				rs1801726							
Secondary malignancy of lymph nodes	0.21	0.52	0.41	0.68	0.44	1.24	3.44	40476	2179	42585	70
Secondary malignancy of respiratory organs	0.53	0.60	0.89	0.37	0.53	1.70	5.50	40476	1249	41656	69
Secondary malignant neoplasm of digestive systems	-11.23	174.62	-0.06	0.95	3.02E-154	1.33E-05	5.84E+143	40476	615	41025	66
Secondary malignant neoplasm of liver	0.37	0.72	0.51	0.61	0.35	1.45	5.98	40476	955	41363	68
Secondary malignancy of brain/spine	0.92	0.72	1.27	0.20	0.61	2.51	10.36	40476	547	40955	68
Secondary malignancy of bone	0.79	0.60	1.32	0.19	0.68	2.20	7.11	40476	958	41365	69
Secondary malignant neoplasm of skin	0.98	1.02	0.96	0.34	0.36	2.65	19.41	40476	259	40668	67

The models were adjusted for median age, sex, and principal components 1-10. Results that passed a significance threshold of $p < 0.01$ are highlighted. None of the results passed Bonferroni correction ($p < 3.38 \times 10^{-4}$). LCI = lower confidence interval; OR = odds ratio; UCI = upper confidence interval.

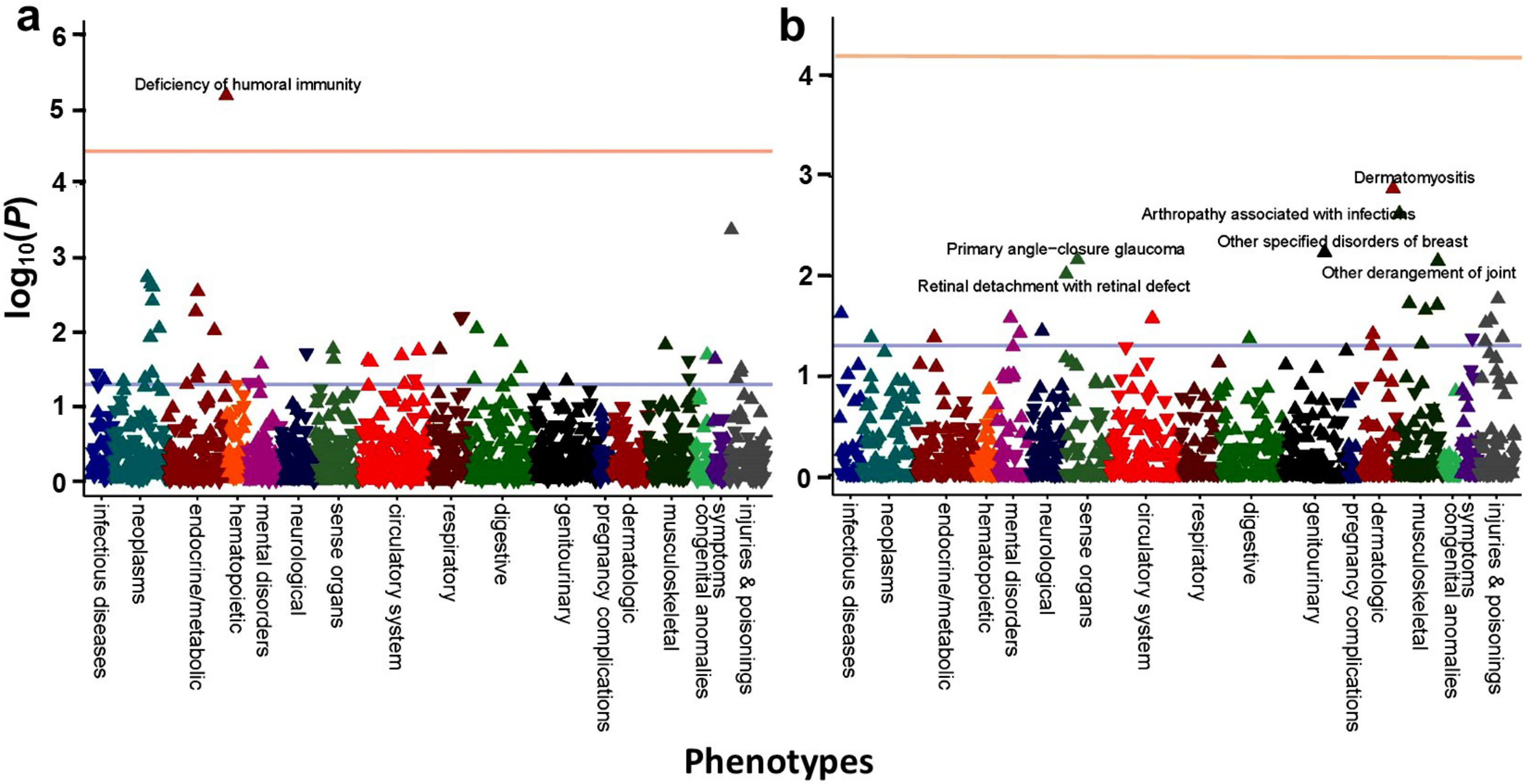

Figure S1. Recessive phenome-wide association study of rs1801725 (A986S) and rs1801726 (Q1011E) in the European descent dataset. The Manhattan plot shows the phenotypes associated with (a) rs1801725 and (b) rs1801276 in a recessive model. Upward arrows represent increased risk and downward arrows indicate decreased risk. None of the phenotypes in (b) passed Bonferroni correction ($p < 3.65 \times 10^{-5}$) represented by the orange line. The blue line represents $p < 0.05$.

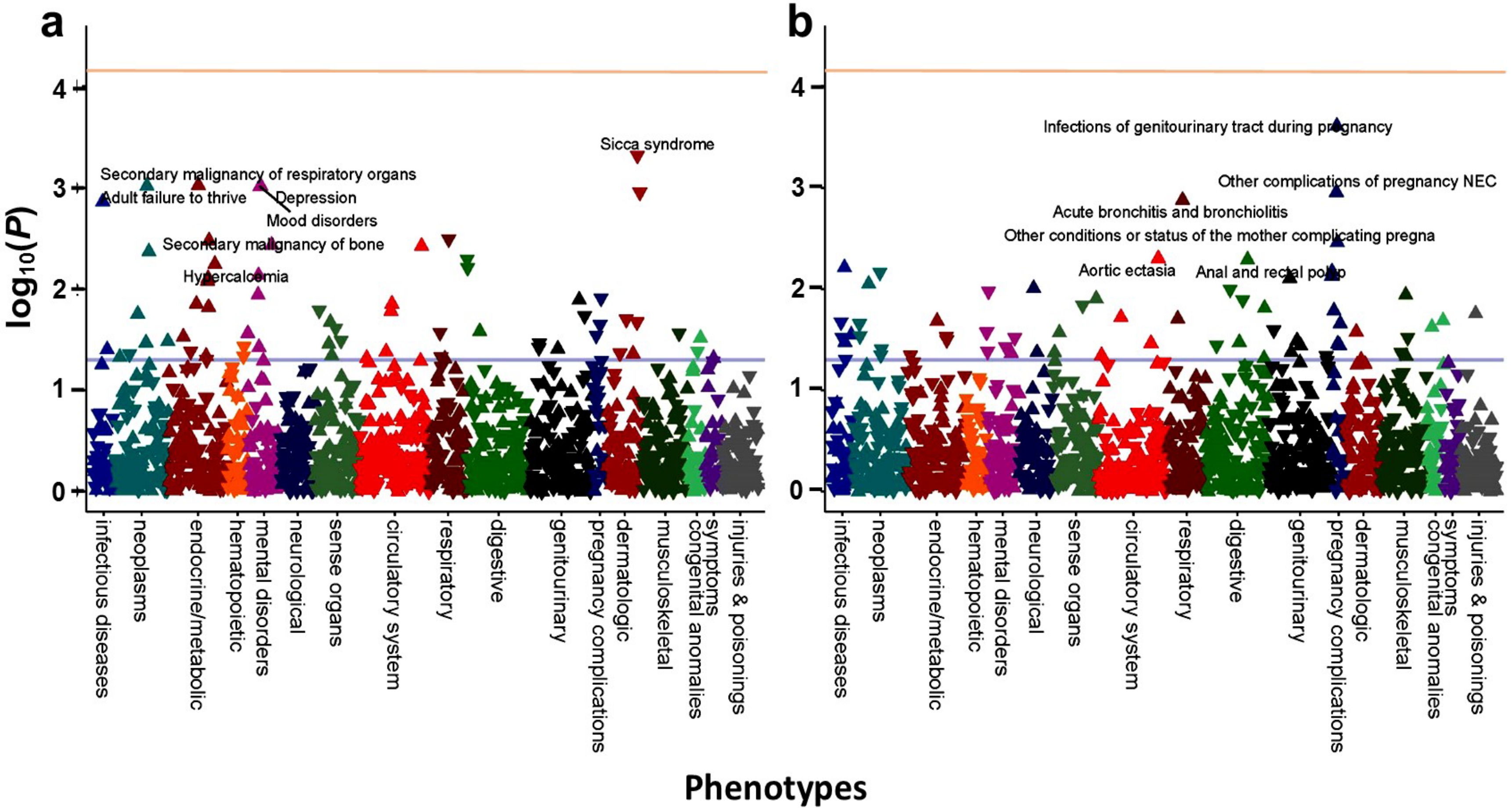

Figure S2. Additive phenome-wide association study of rs1801725 (A986S) and rs1801726 (Q1011E) in the European descent dataset. The Manhattan plot shows the phenotypes associated with (a) rs1801725 and (b) rs1801276 in an additive model. Upward arrows represent increased risk and downward arrows indicate decreased risk. None of the phenotypes passed Bonferroni correction ($p < 3.65 \times 10^{-5}$) represented by the orange line. The blue line represents $p < 0.05$.

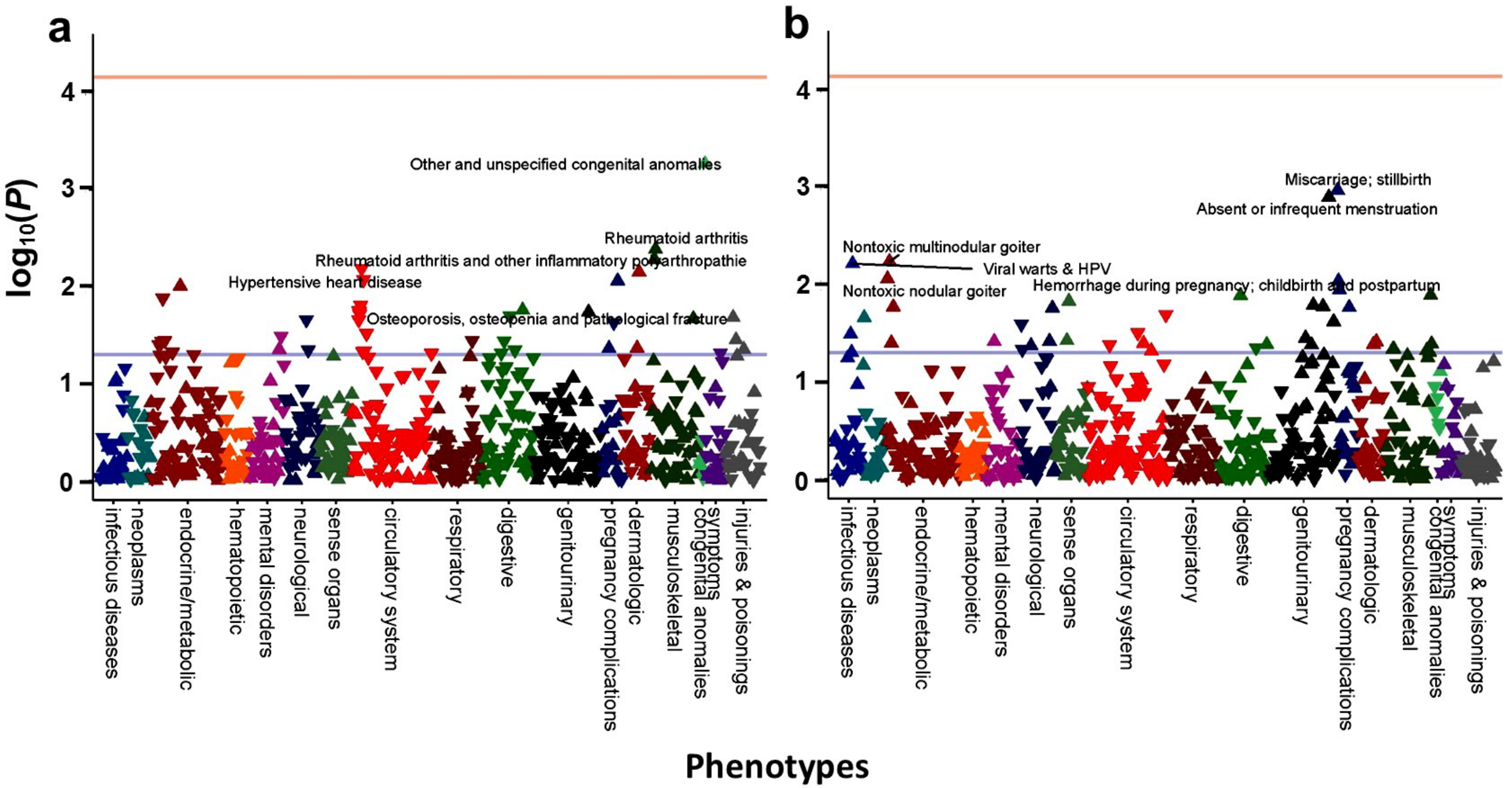

Figure S3. Recessive phenome-wide association study of rs1801725 (A986S) and rs1801726 (Q1011E) in the African descent dataset. The Manhattan plot shows the phenotypes associated with (a) rs1801725 and (b) rs1801276 in a recessive model. Upward arrows represent increased risk and downward arrows indicate decreased risk. None of the phenotypes passed Bonferroni correction ($p < 7.33 \times 10^{-5}$) represented by the orange line. The blue line represents $p < 0.05$.

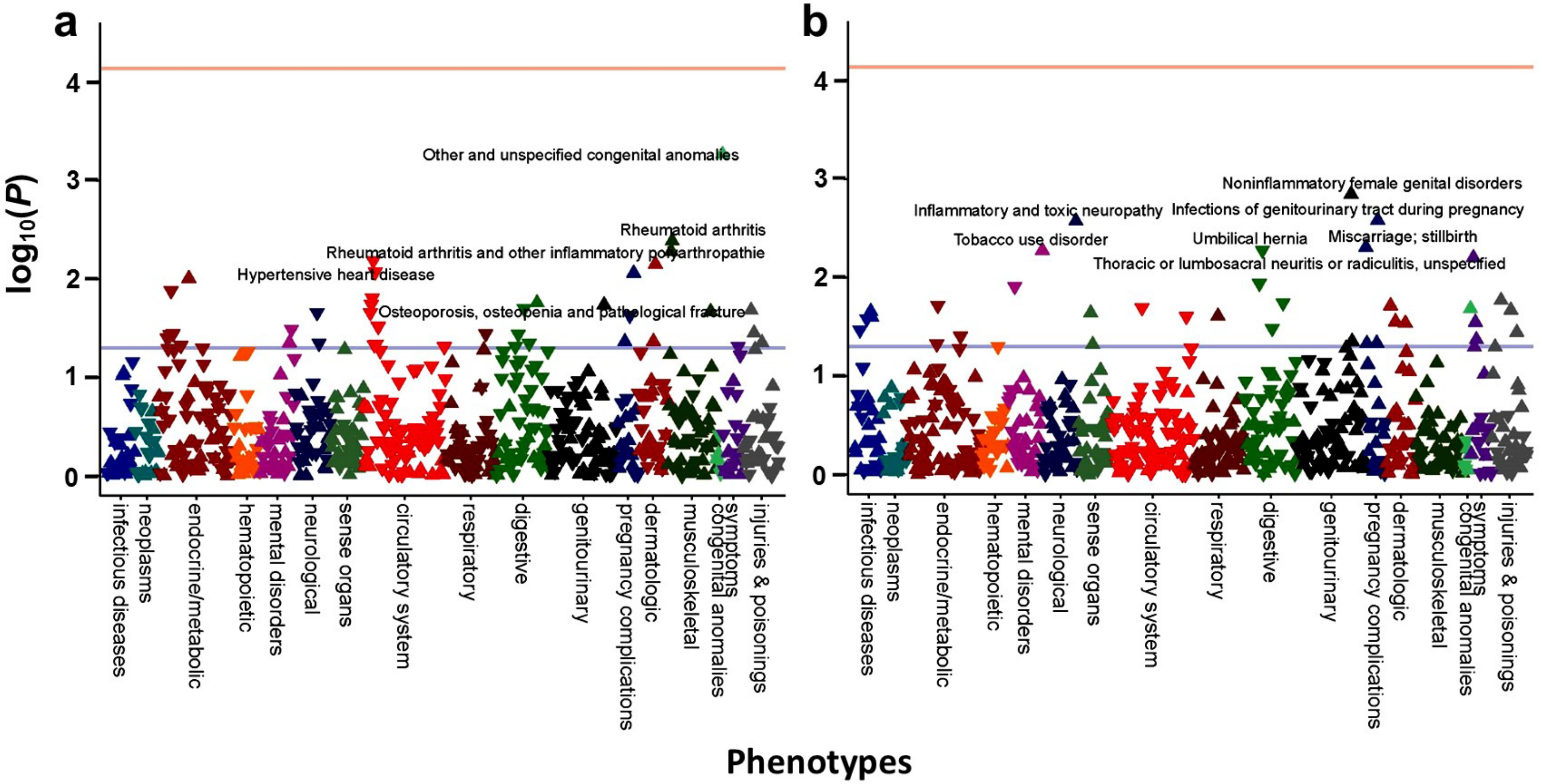

Figure S4. Additive phenome-wide association study of rs1801725 (A986S) and rs1801726 (Q1011E) in the African descent dataset. The Manhattan plot shows the phenotypes associated with (a) rs1801725 and (b) rs1801276 in an additive model. Upward arrows represent increased risk and downward arrows indicate decreased risk. None of the phenotypes passed Bonferroni correction ($p < 7.33 \times 10^{-5}$) represented by the orange line. The blue line represents $p < 0.05$.
